# Computational analysis of radiative engine oil-based Prandtl–Eyring hybrid nanofluid flow with variable heat transfer using the Cattaneo–Christov heat flux model

**DOI:** 10.1039/d2ra08197k

**Published:** 2023-01-25

**Authors:** Zahir Shah, Muhammad Rooman, Meshal Shutaywi

**Affiliations:** a Department of Mathematical Sciences, University of Lakki Marwat Lakki Marwat 28420 Khyber Pakhtunkhwa Pakistan Zahir@ulm.edu.pk; b Department of Mathematics, College of Science & Arts, King Abdul-Aziz University Rabigh Saudi Arabia

## Abstract

In the present analysis, we study the energy transference through engine oil-based Prandtl–Eyring nanofluid flow through a heated stretching surface. The nanofluid is prepared by adding copper (Cu) and titanium dioxide (TiO_2_) nanoparticles (NPs) to the base fluid engine oil. The flow mechanism and thermal transmission are observed by exposing the nanofluid flow through the heated slippery surface. The influences of permeable surface, radiative flux and heat absorption/generation are also elaborated in this study. The flow of nanofluids has been designed using a PDEs system, which are then transformed into a set of ODEs *via* resemblance modification. The numerical technique “shooting method” is used to solve the acquired nonlinear set of non – dimensional ODEs. The results are physically exemplified through tables and plots. It has been detected that the accumulation of nanomaterials in the engine oil, reduces the skin friction while accelerating the energy transfer rate. The velocity field significantly decelerates with the encouragement of the porosity factor, and volume fraction of NPs. However, the temperature profile significantly escalates with the encouragement of the porosity factor, and volume fraction of NPs.

## Abstract 2

In the present analysis, we study the energy transference through engine oil-based Prandtl–Eyring nanofluid flow through a heated stretching surface.

## Graphical abstract

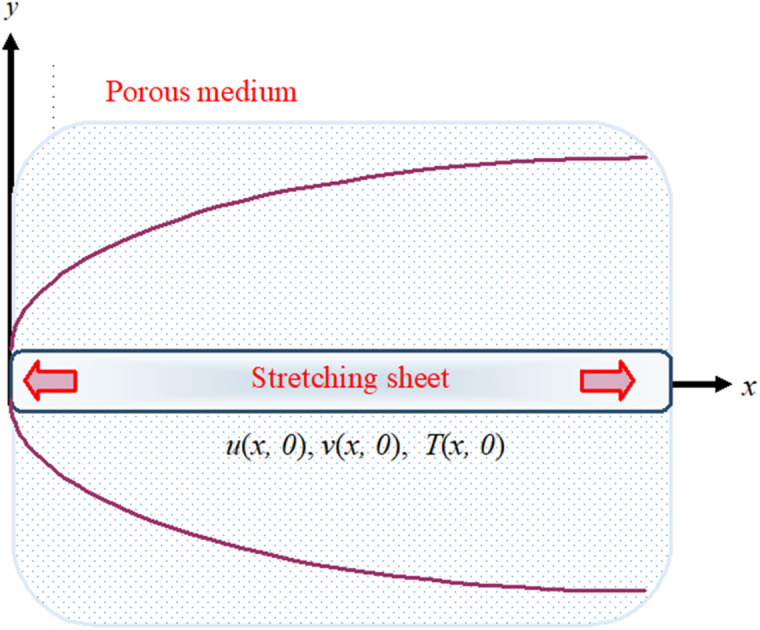

## 1. Introduction

Base fluids (traditional liquids) play a critical role in transferring heat in industrial operations. In general, the heat transferability of these liquids is poor. To overcome this obstacle, nanoparticles (<100 nm) are inserted to enhance abilities of heat transport. Choi and Eastman^1^ proposed this idea first. Solid particles conduct thermal heat more efficiently than liquids, which is a widely accepted fact. Therefore, the addition of particles of nanoscale size to conventional fluids significantly enhanced their thermal conductivity. Nanoparticles is the name given to these solid particles. A nanofluid is a fluid that consists of both the nanoparticles and base fluid. Eastman^2^ claimed in an experimental study that a slight quantity of nanoscale solid substantial particles can enhance the thermal conductivity of normal liquids. This research found that adding Cu NPs or carbon nanotubes (CNTs) at 1% enhanced the thermal efficiency of ethylene glycol (the base fluid) by 40–50% (volume fraction). This is due to nanofluids playing an important part in electromechanical devices, advanced cooling systems, heat exchange, and so on. The stimulus of radiant energy on a hydromagnetic unsteady liquid flow through a leaky stretchable sheet with heat and mass conversion was described by Bilal *et al.*^3^ The micro-rotation characteristic was discovered to be caused by the permeability factor. Ghasemi *et al.*^4^ revealed the influence of magnetic pitch on nanofluid flow across a stretching sheet at the stagnation point. Furthermore, the outcomes revealed that as the Lewis number upsurges, so does the heat flux of the nanofluid. The magnetohydrodynamic water-based NF flow containing motile microorganisms and nanotubes across a porous vertical floating substrate was investigated by Algehyne *et al.*^5^ Increasing heat absorption and production rates were thought to increase the rate of energy transference. Investigators^6–11^ looked into the thermal properties of nanofluids by incorporating multiple types of nanoparticles into the base fluid.

Recently, hybrid nanofluids (HNF), a more advanced type of nanofluid, have been introduced. The hybrid nanofluids are made up of an ordinary liquid and two or more types of nanoparticles. In terms of heat transport, hybrid nanofluids outperform conventional nanofluids. Suresh *et al.*^12^ exercised an experimental two-step method to create a hybrid nanofluid composed of Al_2_O_3_–Cu/water. Madhesh *et al.*^13^ conducted experiments on a copper–titania (Cu–TiO_2_) hybrid nanocomposite and copper–titania (Cu–TiO_2_) HNF flows with volume fraction ranging from 0.1% to 1.0%. The outcomes discovered that for a volume concentration of up to 1%, the heat flux rate is enhanced by 49%. Toghraie *et al.*^14^ carried out a research on the mixture of a ZnO–TiO_2_/EG HNF to illustrate the influences of nanoparticle temperature and concentration on the conduction of the HNF. The outcomes were intriguing, showing that at 50 degrees Celsius, heat radiation was 32% with a volume fraction of 3.5%. In addition to these experimental efforts, scientists have concentrated on theoretical research of hybrid nanoliquid flows. Gul *et al.*^15^ compared Yamada–Ota and Hamilton–Crosser HNF models that contained silicon carbide (SiC) and titanium oxide TiO_2_ nanoparticles (NPs) in diathermic oil. After being stimulated with a magnetic dipole, the flow of the hybrid nanoliquid was predicted to occur over a larger surface. The key findings indicated that the Yamada–Ota model outperformed the Hamilton–Crosser hybrid nanoliquid flow model in terms of heat transfer efficiency. Arif *et al.*^16^ studied theoretically ternary hybrid nanoliquid flow with base liquid water between two parallel sheets with various nanoparticle shapes such as cylinders, spheres, and platelets of carbon nanotubes, aluminium oxide, and graphene, respectively. The unsteady fluid flow and energy transmission of a Cu–Al_2_O_3_/water-based HNF over an axially impermeable contracting and extending substrate were investigated by Khan *et al.*^17^ A hybrid nanofluid (Cu–Al_2_O_3_/water) was discovered to accelerate heat transmission when compared to a conventional fluid. Elattar *et al.*^18^ explored the flow of a steady electrically charged HNF through an opaque thin flexible sheet using the computational technique PCM. The influence of velocity index and Hall current raises the velocity contour, while changes in particle volume and sheet thickness lower it. Wang *et al.*^19^ inspected the influence of a biochemical reaction on a HNF unsteady flow along a texture that was expanding. The presence of the unsteadiness variable has been observed to regulate the transition from laminar to turbulent flow. Alharbi *et al.*^20^ characterised the flow of an energy propagating high conductivity ternary HNF that included nanocrystals as well as an extended sheet. When ternary hybrid NPs are varied the base fluid thermal conductivity is greatly improved. Ahmad *et al.*^21^ looked into the heat transport properties of engine oil containing nanoparticles like Cu and TiO_2_. They found that the effectiveness of copper and titanium oxide in engine oil is overlooked.

Prandtl–Eyring nanofluid is a mixed convection flow of nanoparticles with activation energy that is non-linear. As a result, numerous non-Newtonian fluid models have been offered in the literature. The Prandtl–Eyring fluid is one of them. Darji *et al.*^22^ described visco-inelastic liquid flow boundary layer similarity solutions. Hayat *et al.*^23^ inspected the consequence of magnetohydrodynamics on the flow of a peristaltic dissipative Prandtl–Eyring liquid. According to the results, Akbar^24^ discovered the convective boundary constraints of Prandtl–Eyring liquid flow with peristatic properties. Khan *et al.*^25^ conducted a mathematical computational evaluation of bio convection on PEF. The effects of thermo/phonetic force and Brownian motion on electrically conducting PEF generated by strained shallow were investigated by Abdelmalek *et al.*^26^

Because of the numerous implementations in nanofluid mechanics, investigators are developing a wave-based heat transfer methodology rather than a diffusion operation.^27–29^ Heat transfer is a well-established phenomenon that happens as a consequence of temperature variations between two distinct objects or between components of an identical system. For decades, Fourier's^30^ fundamental law of heat conduction, has been used to measure heat transfer properties. Later, with concern, it was discovered that this model produces a parabolic energy equation with an initial disruption that lasts across the process. A “paradox of heat conduction” denotes this weakness in the Fourier model. Cattaneo^31^ addresses this flaw by incorporating a relaxation term into the Fourier approach. Afterward, Christov^32^ created the Cattaneo-suggested relationship with the Oldroyd upper-convected variant *via* frame-indifferent alteration. This type of relationship is identified as the Cattaneo–Christov (CC) flux model. Kumar *et al.*^33^ used a CC flux model to investigate the features of Dusty fluid of dissolved HNF flows in two phases through an elongated cylinder. The shooting approach with RK–Fehlberg system, were used for numerical results. Ramzan *et al.*^34^ used the CC model and MHD impact with heterogeneous reactions adjacent to a stagnation point to calculate the Williamson fluid flow. It should be acknowledged that the fluid parameter has a diametrically opposed influence on velocity and temperature profiles. Shah *et al.*^35^ used the CC model to investigate heat transfer in a 2D (two dimensional) flow of Ree–Eyring nanoliquid through a stretching sheet.

The primary goal of this research is to determine how heat absorption and thermal radiation, as well as viscous dissipation and energy transportation, occur in engine oil-based NF flow over a heated elongating surface. Cu and TiO_2_ nanoparticles are added to engine oil to create the nanofluid. The nanofluid flow was modelled as a system of PDEs, which are then transformed into a set of ODEs *via* resemblance modification. The numerical technique “shooting method” is used to solve the acquired nonlinear set of non – dimensional ODEs. Local velocity gradient and Nusselt number statistics are estimated and analysed. Despite the fact that the phenomena described in this manuscript have never been attempted before.

## 2. Mathematical formulation

**Fig. 1:**
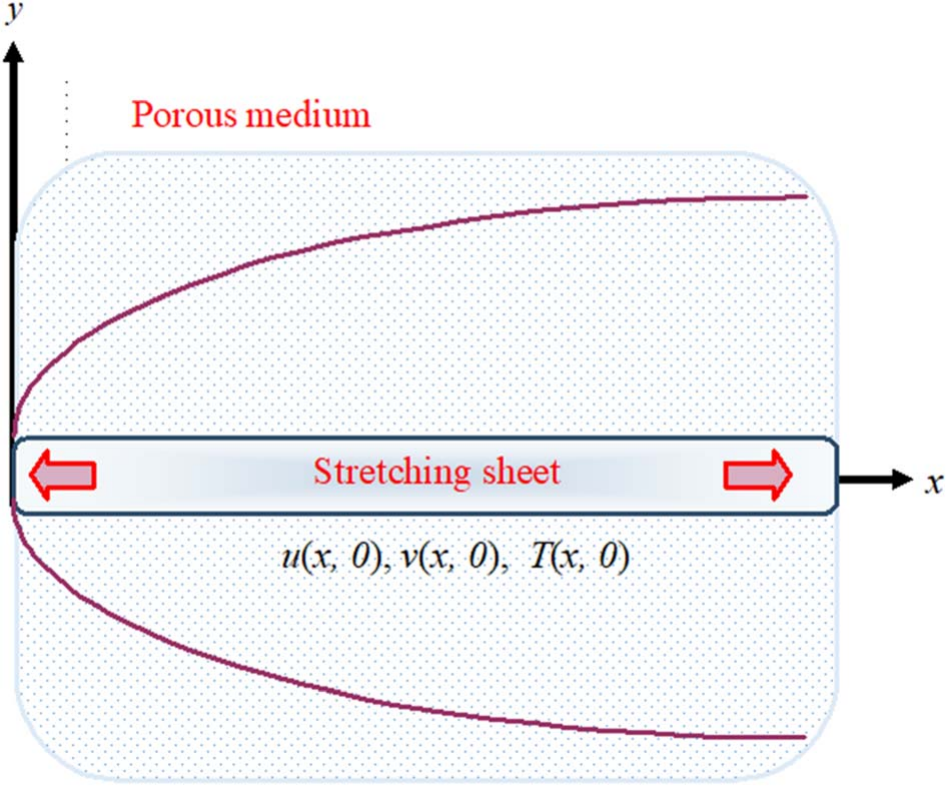
The physical representation of flowing fluid across a stretching surface.

We have assumed the 2D viscous dissipative nanofluid flow over an irregular moving horizontal porous plate. The plate is moving with velocity 
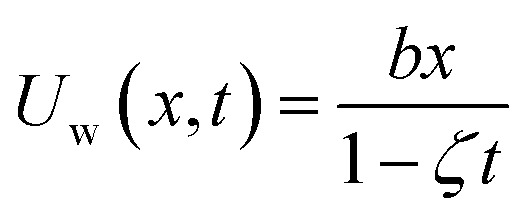
, where *b* is a stretching rate as shown in Fig. 1. The flow has been investigated in terms of thermal radiation and viscous dissipation. At *x* = 0 the surface is heated with relation 
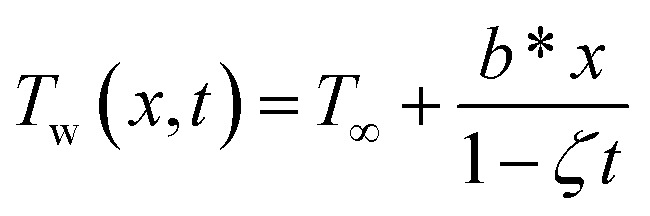
 where b* and *T*_w_ represents the energy variation, surrounding temperature and surface heat, respectively. The surface of the plate is supposed to be slippery.

### 2.1. Formal model

The fluid flow model is displayed in Fig. 1 as:

### 2.2. Governing equations

The constitutive Prandtl–Eyring model^33–35^ for the nanofluid flow in a porous medium and heat equation with variable temperature, thermal radiation, Cattaneo–Christov heat flux model, and heat source/sink utilizing the approximate boundary-layer are:1
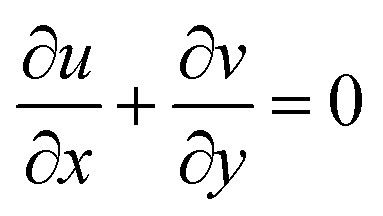
2
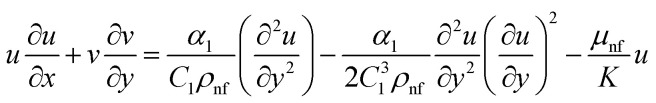
3
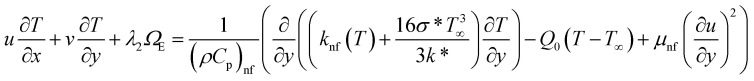


Boundary conditions^21^4
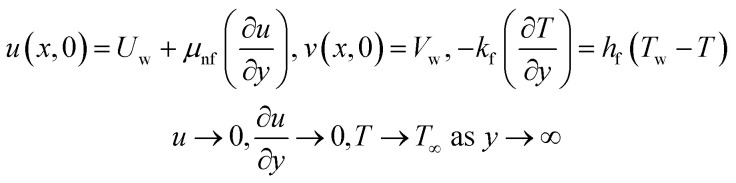
Here, *v*, and *u* are the velocity components, *α*_1_ and *C*_1_ are the fluid parameters, *μ*_nf_ is the dynamic viscosity of nanofluid, *ρ*_nf_ is the density, *k*_nf_ is the thermal conductivity, *Q*_0_ is the heat source, *h*_f_ is the heat transition constant and *K* is the surface permeability, *Ω*_E_ is the heat flux for which model equations is5
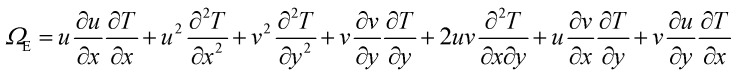


The varying thermal conductivity is classified as:6
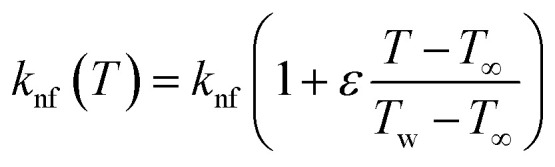


### 2.3. Thermo-physical material properties of nanofluid and base fluid

Different researchers presented mathematical models that explain the effective characteristics of heat transfer in the nanofluids. These models present physical characteristic of the nanofluid in term of relevant physical characteristics of the solid nanoparticles and base fluid. The density of a ferrofluid (*ρ*_ff_) is related to the density of the fluid (*ρ*_f_) and that of the solid nanoparticle phase (*ρ*_s_) as follows:^35^

7 *ρ*_nf_ = (1 − *ϕ*)*ρ*_f_ + *ϕρ*_s_

Here *ϕ* is the volume fraction of NPs. Similarly the volume specific heats are correlated as:

8 (*ρC*_p_)_nf_ = (1 − *ϕ*)(*ρC*_p_)_f_ + *ϕ*(*ρC*_p_)_s_

The dynamic viscosity of the fluid and the nanofluid are given by;

9 *μ*_nf_ = (1 − *ϕ*)^−2.5^*μ*_f_

To estimate the efficient thermal conductivity of the nanofluid, the Maxwell–Garnetts (MG) model can be utilized:10
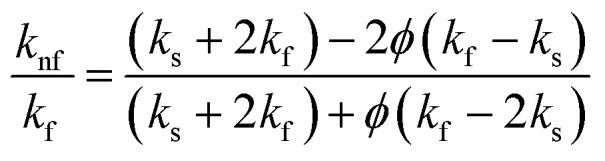


In the present study heat transfer analysis through copper (Cu) and titanium dioxide (TiO_2_) nanoparticles (NPs) to the base fluid engine oil has been achieved into the account the characteristics using in Table 1.

**Table 1:** The experimental values of engine oil, Cu and TiO_2_ (ref. 21)

	*ρ* (kg m^−3^)	*C*_p_ (j kg^−1^ K^−1^)	*k* (W mK^−1^)
Engine oil	884	1910	0.1440
Copper Cu	8933	385.0	401.0
Titanium dioxide TiO_2_	4250	686.20	8.9538

## 3. Dimensionless formulations model

Using all assumptions and velocity filed on eqn (1)–(4), eqn (1) hold identically, and the dimensionless process for eqn (2)–(4), the stream functions are expressed as:11
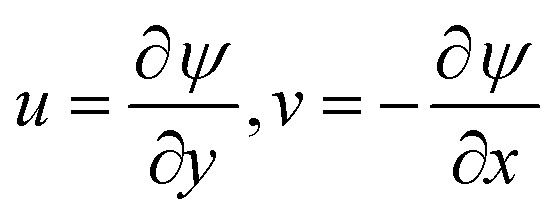


The similarity variables are:12
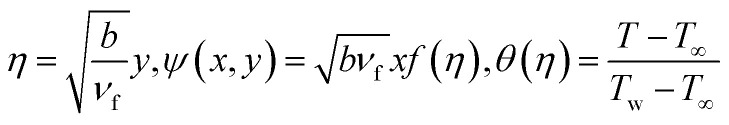


Using eqn (8) & (9) in eqn (1)–(3), we get the following dimensionless form of a system of ODEs:13
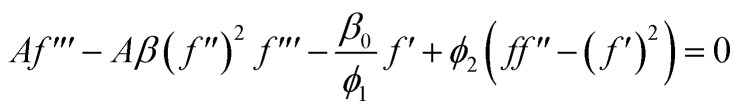
14
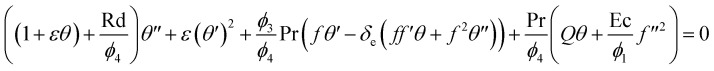


The transform boundary conditions are:15
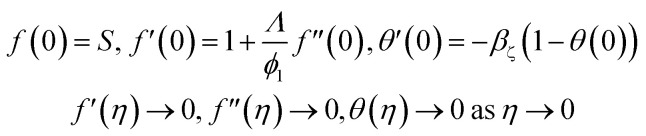
where,16
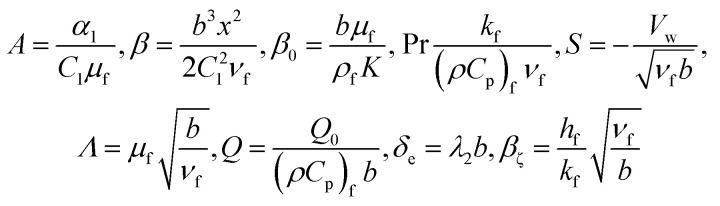


Also17
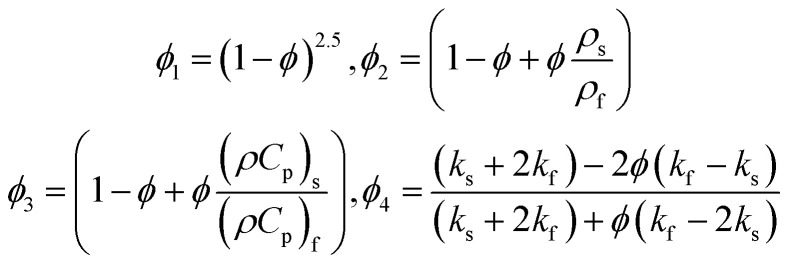


## 4. Skin friction drag and nusselt number

The physical interest quantities such as the drag force *C*_f_ and nusselt number Nu are specify as18
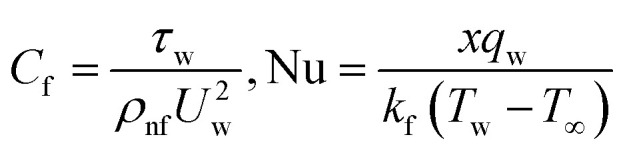
where19
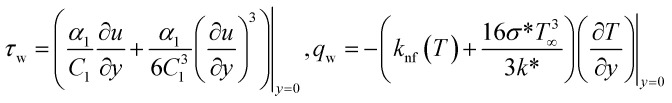


Incorporating eqn (15) in (14), we get:20
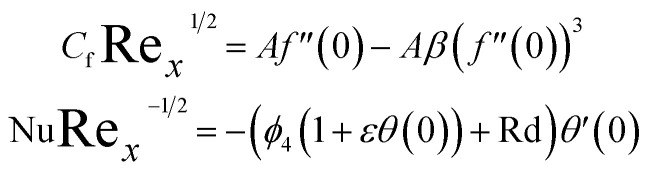


## 5. Numerical solution

The obtained system of ODEs (eqn (13)–(15)) is further reduced to the 1^st^ order differential equations through the following variables framework:

21 *f* = *¥*_1_, *f*′ = *¥*_2_, *f*′′ = *¥*_3_, *θ* = *¥*_4_, *θ*′ = *¥*_5_

22
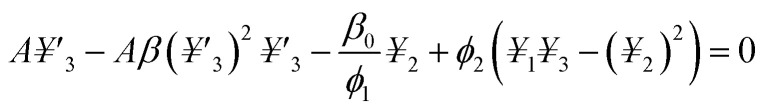
23
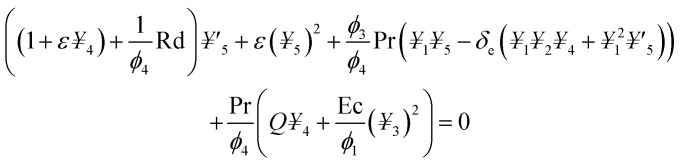


The transform boundary conditions are:24
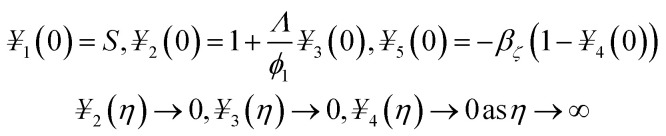


## 6. Result and discussion

This section explained the physical procedure and trend that underpin each plot and table. The physical sketch of the flow problem was elaborated in Fig. 1. Fig. 2–13 show the behaviour of velocity and energy outlines in relation to various physical constraints.

### 6.1. Velocity profile

**Fig. 2:**
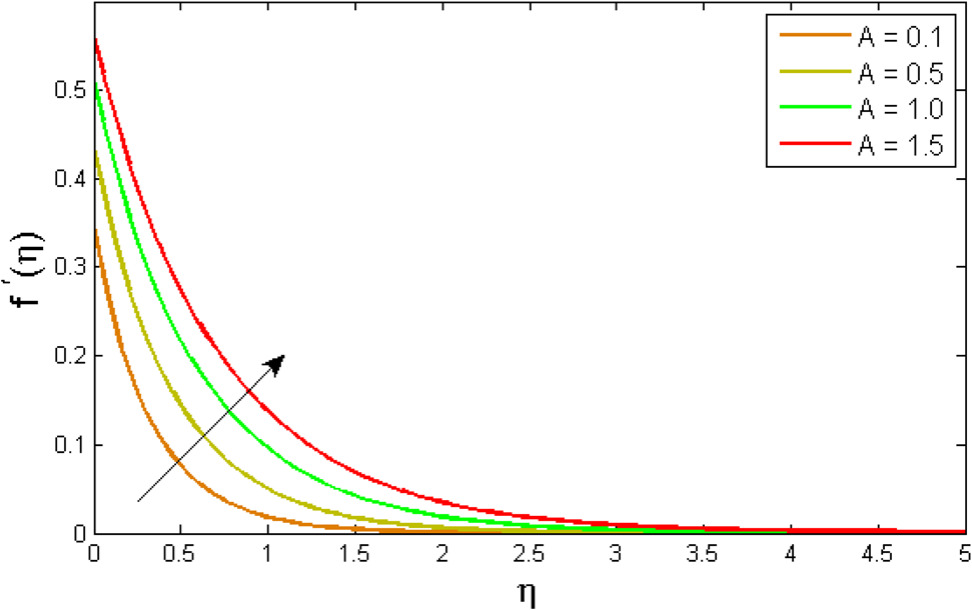
Variation of velocity outlines *f*′(*η*) *versus* fluid parameter *A*.

**Fig. 3:**
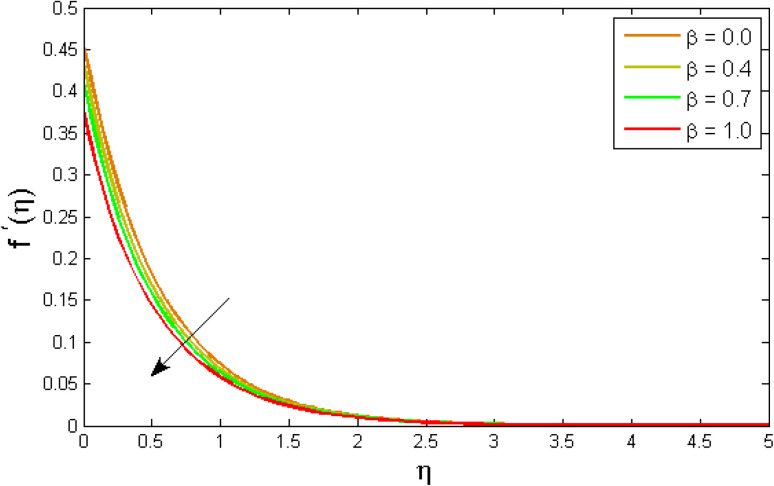
Variation of velocity outlines *f*′(*η*) *versus* fluid parameter *β*.

**Fig. 4:**
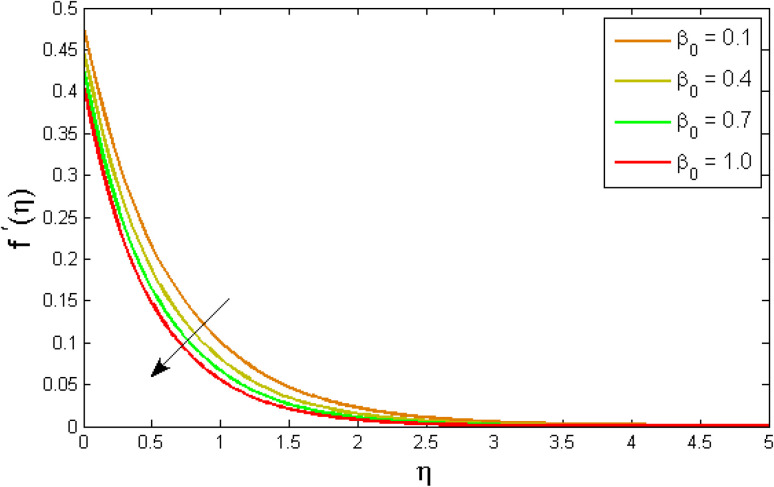
Variation of velocity outlines *f*′(*η*) *versus* porosity parameter *β*_0_.

**Fig. 5:**
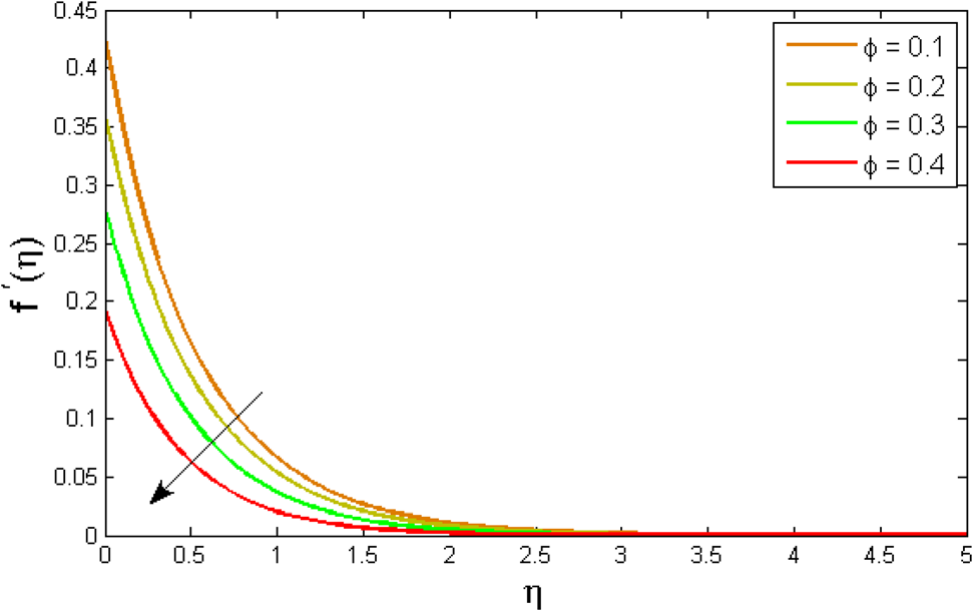
Variation of velocity outlines *f*′(*η*) *versus* volume fraction parameter *ϕ*.

**Fig. 6:**
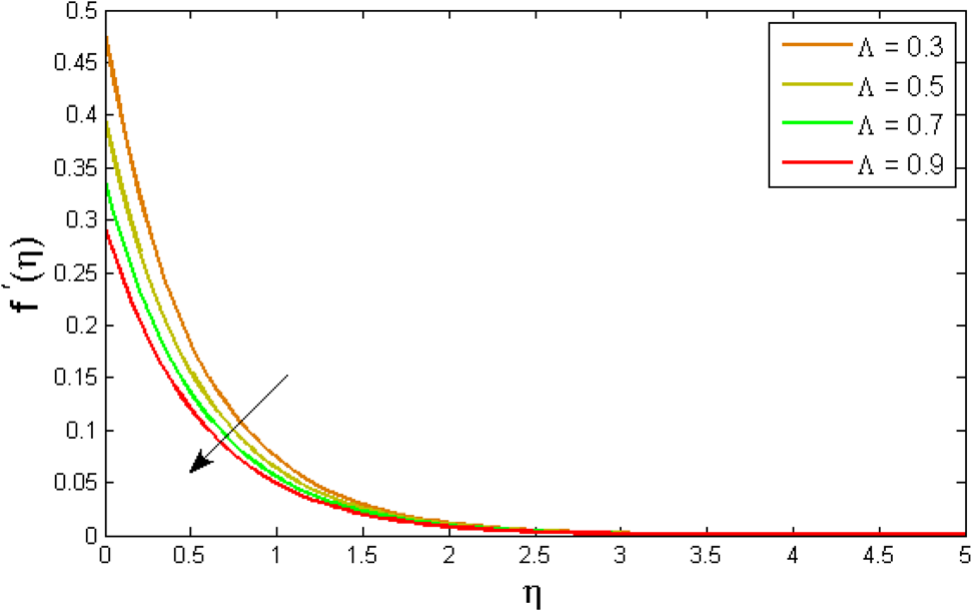
Variation of velocity outlines *f*′(*η*) *versus* velocity slip parameter *Λ*.

**Fig. 7:**
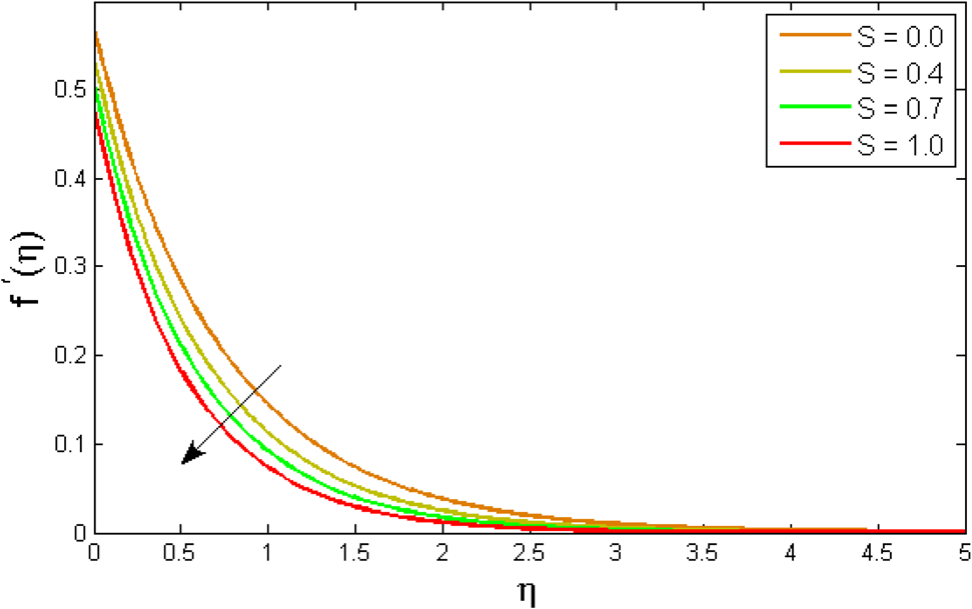
Variation of a velocity outlines *f*′(*η*) *versus* mass transfer parameter *S*.

Fig. 2–7 elucidated the trend of velocity outlines *f*′(*η*) *versus* fluid parameters *A* & *β*, porosity parameter *β*_0_, velocity slip parameter *Λ*, volume fraction parameter *ϕ*, and suction parameter *S*. Fig. 2 illustrates the effects of fluid parameter *A* on fluid velocity while the other parameters are held constant. This graph shows that increasing the value of *A* causes an upsurge in the value of velocity. Because higher values of *A* tend to reduce viscosity, which overwhelms the resistance offered by the liquid. Fig. 3 depicts how the fluid velocity gradient tends to decrease as fluid parameter *β* increases. It is physically true because *β* varies inverse proportion with momentum diffusivity, resulting in a decrease in velocity gradient. Fig. 4 portrays the impacts of the porosity parameter *β*_0_ on the velocity distribution. The velocity decreases as *β*_0_ increases. Physically, the existence of a porous medium has increased the medium's opposition to fluid flow. Fig. 5 shows how the volumetric concentration *ϕ* affects the velocity profile *f*′(*η*). When the particle volume *ϕ* fraction is risen, the velocity profile *f*′(*η*) reduces. As the volume fraction *ϕ* of the nanoparticles grows, the fluid thickens, and a conflicting force develops, leading to deceleration. Fig. 6 depicts a graphical representation of the behaviour of velocity profiles *f*′(*η*) as a function of the velocity slip parameter *Λ*. In general, *Λ* calculates the amount of slip at the cylinder's surface. Here, we examine how fluid velocity decreases as *Λ* increases. It is because *Λ* primarily reduce speed of fluid motion, confirming a reduction in net movement of fluid molecules. Because there is less molecular progression, velocity fields decline. Fig. 7 indicates the behaviour of the velocity field for various values of *S*. Suction is an efficient method for preventing boundary layer separation, as well as controlling velocity and heat energy. The amount of fluid particles is close to the wall after reaching the maximum value of the suction/blowing parameter. Accordingly, the outline of the associated boundary layer becomes thinner over time, and the velocity profiles decelerate as S strength increases.

### 6.2. Temperature profile

**Fig. 8:**
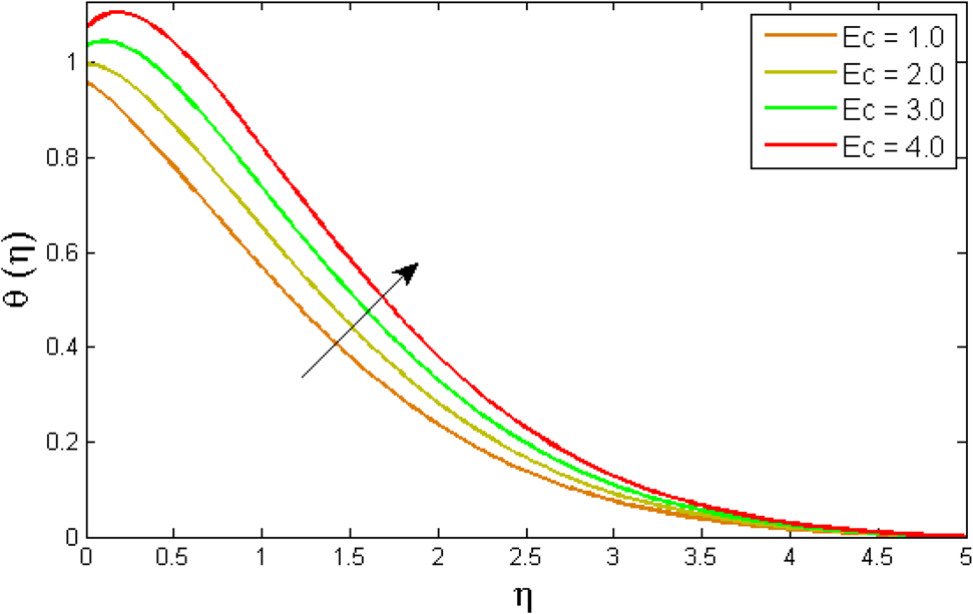
Variation of temperature outlines *θ*(*η*) *versus* Eckert number Ec.

**Fig. 9:**
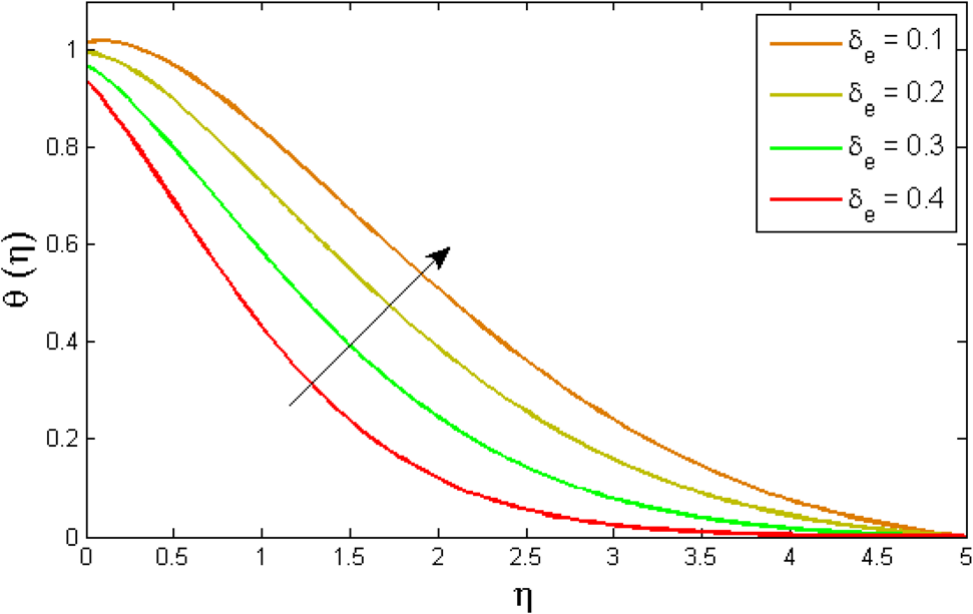
Variation of temperature outlines *θ*(*η*) *versus* thermal relaxation parameter *δ*_e_.

**Fig. 10:**
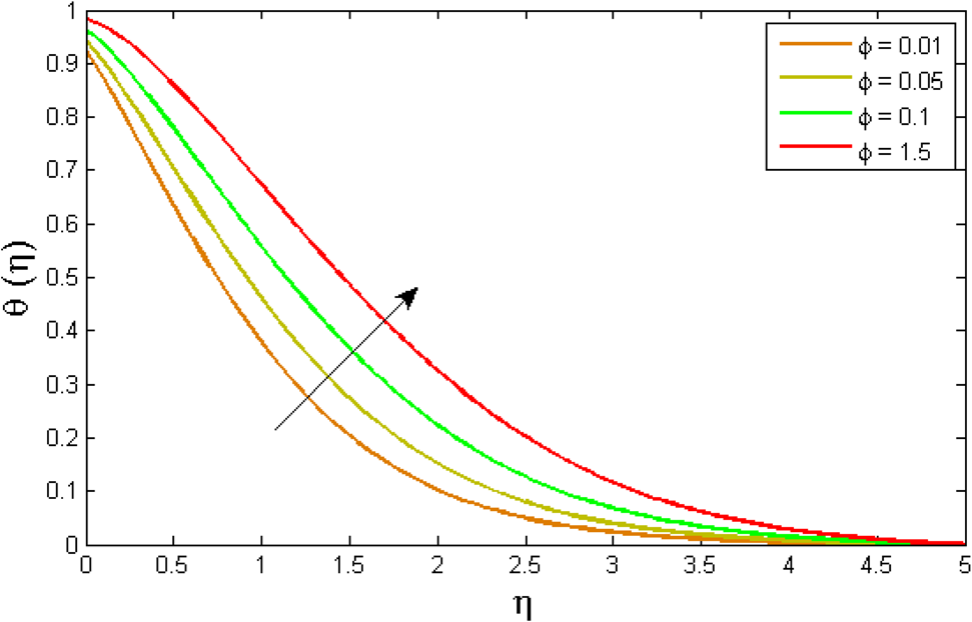
Variation of temperature outlines *θ*(*η*) *versus* volume fraction parameter *ϕ*.

**Fig. 11:**
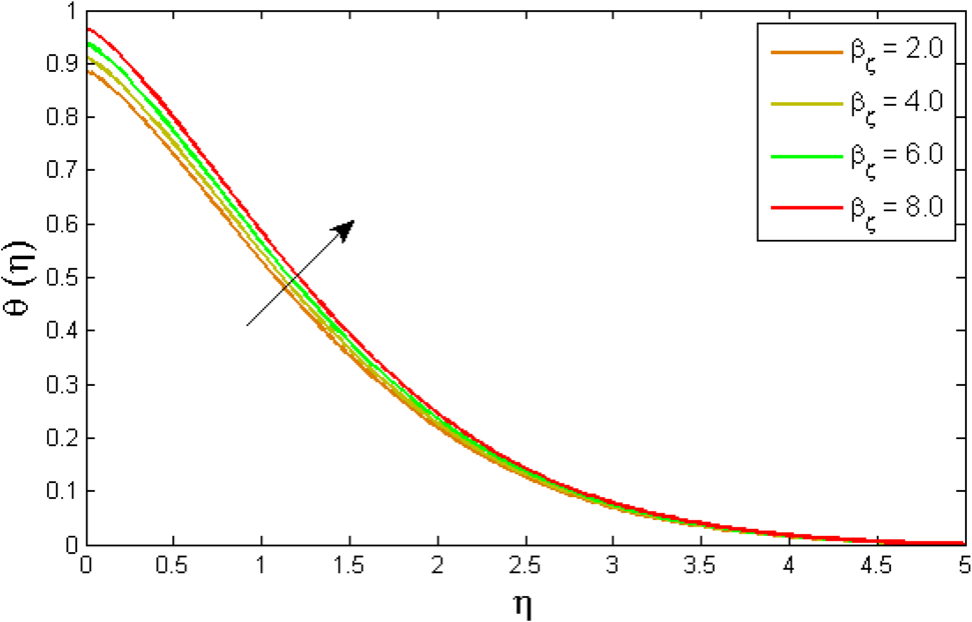
Variation of temperature outlines *θ*(*η*) *versus* Biot number *B*_ζ_.

**Fig. 12:**
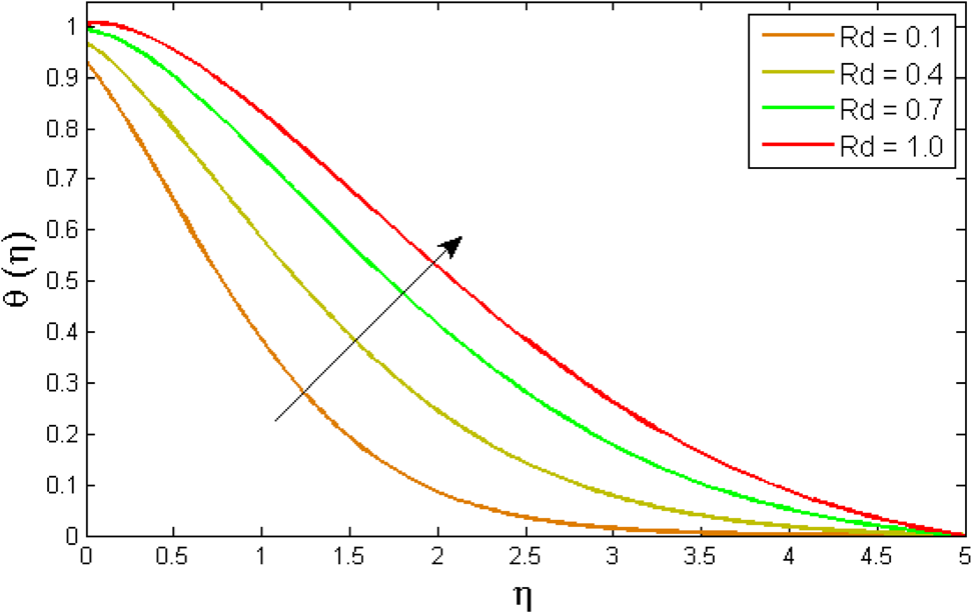
Variation of temperature outlines *θ*(*η*) *versus* temperature dependent thermal radiation parameter Rd.

**Fig. 13:**
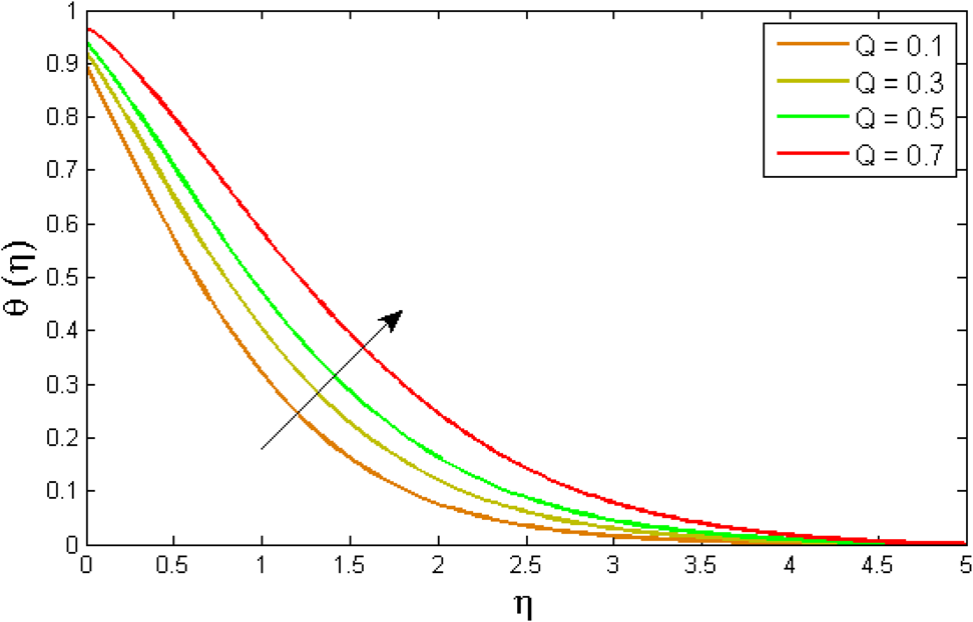
Variation of temperature outlines *θ*(*η*) *versus* heat generation parameter *Q*.

Fig. 8–13 reported the conduct of the energy profile *θ*(*η*) *versus* the thermal relaxation parameter *δ*_e_, volume fraction parameter *ϕ*, Biot number *B*_ζ_, heat generation constraint *Q*, thermal radiation term Rd and Eckert number Ec. Fig. 8 depicts that the variation of Eckert number Ec on temperature profile. The stimulus of Eckert number Ec on nanofluid temperature is evident because an increase in Eckert number accelerates advective transport (kinetic energy). As a result, fluid particles interact together more frequently, and these collisions convert kinetic energy (KE) into thermal energy. Accordingly, the temperature profile upsurges. Fig. 9 displays the stimulus of the thermal relaxation parameter on temperature distribution. Temperature distribution decreases as the thermal relaxation parameter *δ*_e_ increases. It is also observed that the thickness of the thermal boundary layer diminutions. This is due to the fact that as *δ*_e_ intensifies, the material particles necessitate more time to transferal heat to their neighbouring droplets. To put it another way, for advanced values of the *δ*_e_ parameter, the material reveals a non-conducting property, which contributes to a narrower temperature distribution. Fig. 10 exposed that the temperature upsurges with the upshot of the volume fraction parameter *ϕ*. Because of the collision of tiny nanoparticles in the flow field produces thermal energy, which advances the temperature of the fluid. Therefore, the addition of Cu and titanium NPs enhances the energy propagation *θ*(*η*). Fig. 11 display the impression of Biot number *B*_ζ_ on the energy profile. The Biot number is related to the surface's convective boundary constraints. As *β*_ζ_ boosts, so does the temperature profile close to the surface, raising the temperature close to the surface and, consequently, the thickness of the thermal boundary layer, as realized in Fig. 11. Fig. 12 indicates that the variation of thermal radiation Rd on temperature profile. The temperature profile improves as thermal radiation (Rd) values boost. Larger Rd values have dominant impact on conduction. As a result of the radiation, a significant quantity of heat is distributed into the system, raising the temperature. Fig. 13 depicts the encouragement of a heat generation factor *Q* on fluid temperature. The temperature profile is seen to boost as *Q* goes up. Heat is generated in the flow regime as a result of positive modifications in the heat generation parameter, causing a rise in fluid temperature.

## 7. Skin friction and nusselt number

Table 2 and 3 quantitively reported the computational outcomes of skin friction *C*_f_Re_*x*_^1/2^ and Nusselt number NuRe_*x*_^−1/2^*versus* different physical entities. It has been perceived that the accumulation of NPs to the engine oil, accelerates the skin friction, while reducing the energy transfer rate. Where, the fluid parameter *β* boost the skin friction, while the variation of porosity parameter dimmish the skin friction as observed in Table 2. It can be noticed from Table 3 that the rising quantity of thermal relaxation parameter, Eckert number and thermal radiation declined the energy transmission rate. In Table 4 the presents results are compared with previous published results, and excellent agreement is noticed in both results.

**Table 2:** :Outputs of skin friction *versus* physical constraints

*β*	*β*_0_	*ϕ*	*C*_f_Re_*x*_^1/2^ for TiO_2_	*C*_f_Re_*x*_^1/2^ for Cu
0.1			−0.8646406	−0.9474403
0.3			−0.7848349	−0.830342
0.5			−0.6975074	−0.6999307
	0.1		−0.8646406	−0.9474403
	0.3		−0.8748317	−0.9533525
	0.5		−0.8840958	−0.9589156
		0.1	−0.8646406	−0.9474403
		0.2	−0.8034606	−0.8956594
		0.3	−0.6906621	−0.7638494

**Table 3:** Outputs of Nusselt number NuRe_*x*_^−1/2^*versus* physical entities

*δ*_e_	Ec	*ϕ*	Rd	NuRe_*x*_^−1/2^ for TiO_2_	NuRe_*x*_^−1/2^ for Cu
0.1				0.7661464	0.6494773
0.3				1.007504	0.8727002
0.5				1.275255	1.132698
	0.1			0.7661464	0.6494773
	0.3			0.686729	0.5710788
	0.5			0.6076494	0.4929863
		0.1		0.7661464	0.6494773
		0.2		0.4552573	0.4717461
		0.3		0.4482464	0.3758579
			0.1	0.7661464	0.6494773
			0.2	0.731588	0.6073274
			0.3	0.6974548	0.5682502

**Table 4:** Comparison of *f*′′(0) for different values of *Λ* with literature Ahmad *et al.*^21^

*Λ*	*f*′′(0) for TiO_2_	*f*′′(0) for Cu	*f*′′(0) for TiO_2_	*f*′′(0) for Cu
	Present paper		Ahmad *et al.*^21^	
0.2	−0.8676146	−0.9223163	−0.82123	−0.92408
0.4	−0.6956203	−0.7309784	−0.67790	−0.74650
0.6	−0.584262	−0.6094886	−0.58010	−0.62984
0.8	−0.5055081	−0.5246363	−0.50856	−0.54659
1.0	−0.446524	−0.4616436	−0.45368	−0.48387

## 8. Conclusions

We analyzed the energy transference and entropy generation through nanofluid flow through a heated extending surface. The nanofluid is organized by the accumulation of Cu and TiO_2_ NPs in the engine oil. The flow of nanofluids has been designed using a PDEs system, which are then transformed into a set of ODEs *via* resemblance modification. The numerical technique “shooting method” is used to solve the acquired nonlinear set of non – dimensional ODEs. The outcomes are physically exemplified through figures and tables. The key findings are:

• The velocity *f*′(*η*) significantly improves with the influence of parameters *A*, while declining with the effects of parameter *β*, suction parameter *S*, slip parameter *Λ*, porosity factor, and volume fraction of nanoparticles.

• The temperature *θ*(*η*) curve boosts with growing values of the thermal relaxation coefficient *δ*_e_ and volume fraction parameter *ϕ*.

• The temperature *θ*(*η*) pointedly boosts with the stimulus of *Q*, Eckert number Ec, and thermal radiation parameter Rd.

• The accumulation of nanomaterials in the engine oil, speeding up the skin friction, while reduces the energy transfer rate.

• The fluid parameter *β* boost the skin friction, while the variant in porosity parameter dimmish the skin friction.

• The rising quantity of thermal relaxation parameter, Eckert number and thermal radiation declined the energy transmission rate.

## Nomenclature

Br: Brinkman number
*C*_p_
: Specific heat transfer (J/Kg/K)
*c*_b_
: Drag force
Ec: Eckert number
*h*_f_
: Heat transition constant
*K*
: Permeability of porous medium
*k*
: Thermal conductivity (W m^−1^ K^−1^)
Pr: Prandtl number
*Q*_0_
: Heat generation/absorption
Rd: Thermal radiation
*T*
: Temperature of fluid
*T*_r_
: Temperature ratio
*u*, *v*: Velocity components (m s^−1^)
*x*, *y*: Coordinates axis (m)

### Greek letter

*α*_1_, *C*_1_: Fluid parameter
*A*, *β*: Dimensionless fluid parameter
*β*_0_
: Porosity parameter
*β*_ζ_
: Biot number
Λ
: Velocity slip parameter
*δ*_e_
: Thermal relaxation parameter
ϕ
: Volume fraction parameter
ε
: Temperature dependent thermal conductivity parameter
η
: Independent coordinate
ν
: Kinematic viscosity
μ
: Dynamic viscosity
ρ
: Density (kg m^−3^)
*ρC*_p_
: Heat capacitance
ψ
: Stream function (m^2^ s^−1^)

### Subscripts

*f*
: Base fluid
*s*
: Solid nanoparticle
nf: Nanoparticles
*w*
: Wall
∞: Free-stream

## Data availability

The data that support the findings of this study are available from the corresponding author upon reasonable request.

## Author contributions

All authors have equal contributions.

## Conflicts of interest

The authors declare that they have no competing interests.

## Supplementary Material
